# Supplementary material for: Isotropic reconstruction for electron tomography with deep learning
Source: Nat Commun. 2022 Oct 29;13:6482. doi: 10.1038/s41467-022-33957-8 (PMC9617606; doi:10.1038/s41467-022-33957-8)
Supplement: Supplementary file 1 — Supplementary Information [file 41467_2022_33957_MOESM1_ESM.pdf]

Supplementary Information for

Isotropic Reconstruction for Electron Tomography with Deep Learning

Yun-Tao Liu[1,2,3,†], Heng Zhang[1,4,†], Hui Wang[2,3,5], Chang-Lu Tao[1,6,7], Guo-Qiang Bi[1,6,8,*] and Z. Hong Zhou[2,3,5,*]

[1]Center for Integrative Imaging, Hefei National Research Center for Physical Sciences at the Microscale, School of Life Sciences, Division of Life Sciences and Medicine, University of Science and Technology of China, Hefei, Anhui 230026, China;
[2]California NanoSystems Institute, University of California, Los Angeles (UCLA), Los Angeles, CA 90095, USA;
[3]Department of Microbiology, Immunology and Molecular Genetics, UCLA, Los Angeles, CA 90095, USA;
[4]Department of Physics, University of Science and Technology of China, Hefei, Anhui 230026, China;
[5]Department of Bioengineering, UCLA, Los Angeles, CA 90095, USA;
[6]Interdisciplinary Center for Brain Information, Brain Cognition and Brain Disease Institute, Shenzhen-Hong Kong Institute of Brain Science-Shenzhen Fundamental Research Institutions, Shenzhen Institute of Advanced Technology, Chinese Academy of Sciences, Shenzhen, 518055, China;
[7]Faculty of Life and Health Sciences, Shenzhen Institute of Advanced Technology, Chinese Academy of Sciences, Shenzhen, 518055, China;
[8]Center for Excellence in Brain Science and Intelligence Technology, Chinese Academy of Sciences, Shanghai 200031, China

[†]These authors contributed equally: Yun-Tao Liu, Heng Zhang.

[*]Correspondence and requests for materials should be addressed to: Z.H.Z: Email: Hong.Zhou@UCLA.edu; or G.-Q. B: gqbi@ustc.edu.cn;

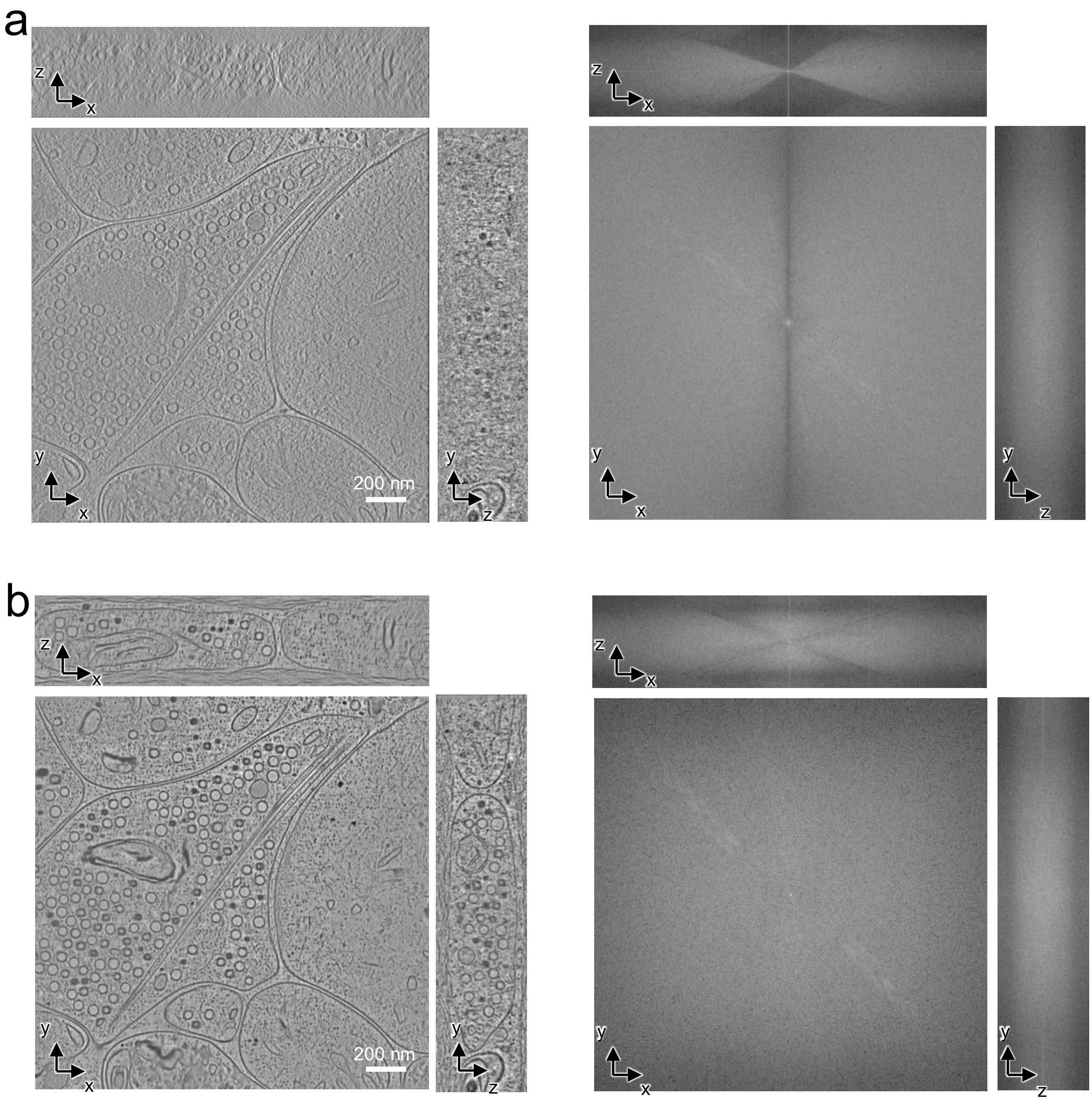

Supplementary Fig. 1│a, Orthogonal views of the WBP reconstructed tomograms and their corresponding Fourier transforms. **b,** Orthogonal views of the IsoNet reconstructed tomograms and their corresponding Fourier transforms.

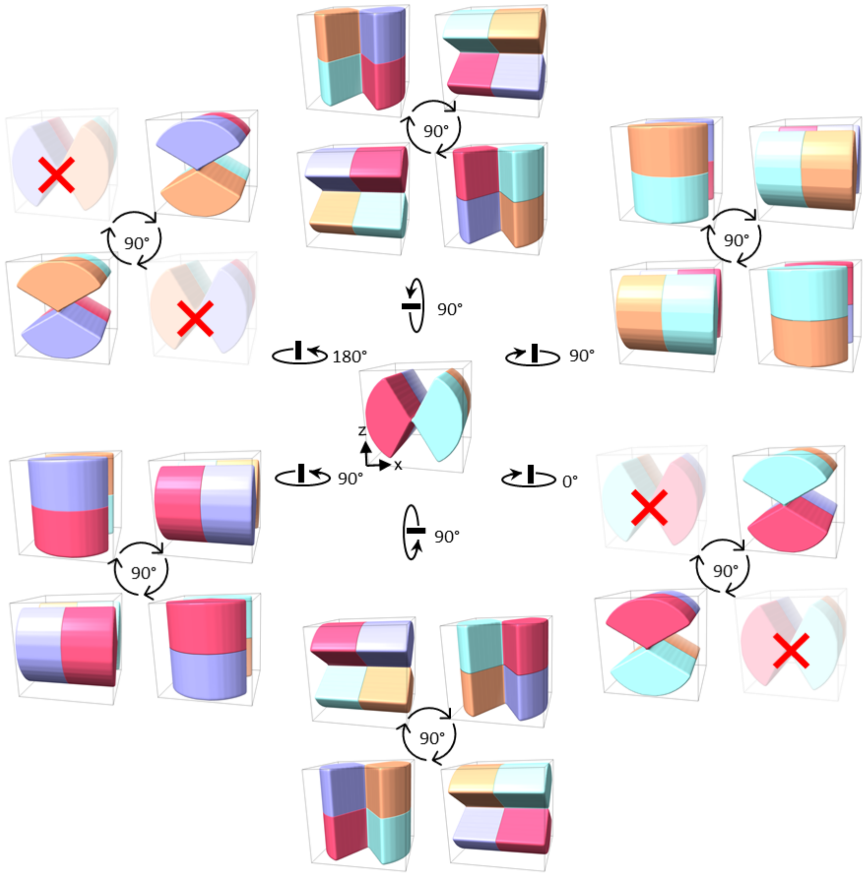

Supplementary Fig. 2 │ Rotation schemes. Twenty rotated copies are obtained for each extracted subtomograms demonstrated in the center. First, each subtomogram has six faces. Each face can be rotated with an out-of-plane angle to face toward six positive directions of the Y-axis. Second, each out-of-plane rotation is followed by four in-plane rotations, making a total of 24 possible rotations. However, among the 24 rotations copies, four have the same Z-axis missing-wedge as the original subtomograms. Thus, these four rotations are excluded (red cross).

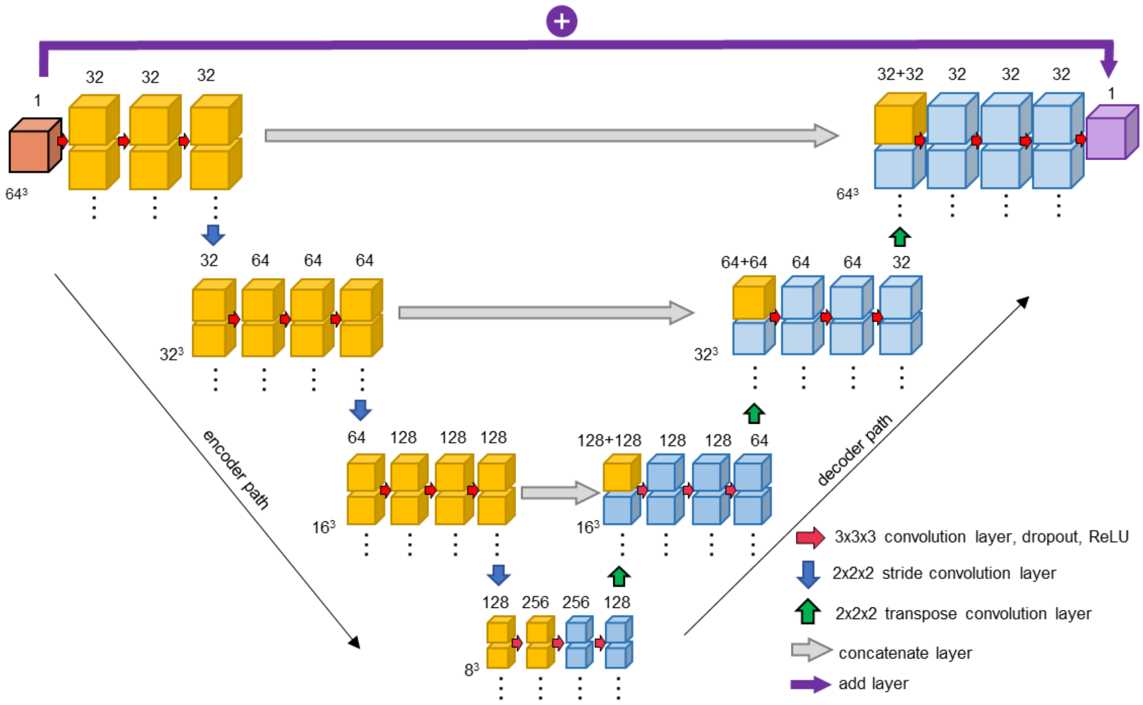

Supplementary Fig. 3 │The architecture of neural network based on U-net. The values at the bottom left of boxes show sizes of 3D feature maps or subtomograms, while the values on top of the boxes are their numbers.

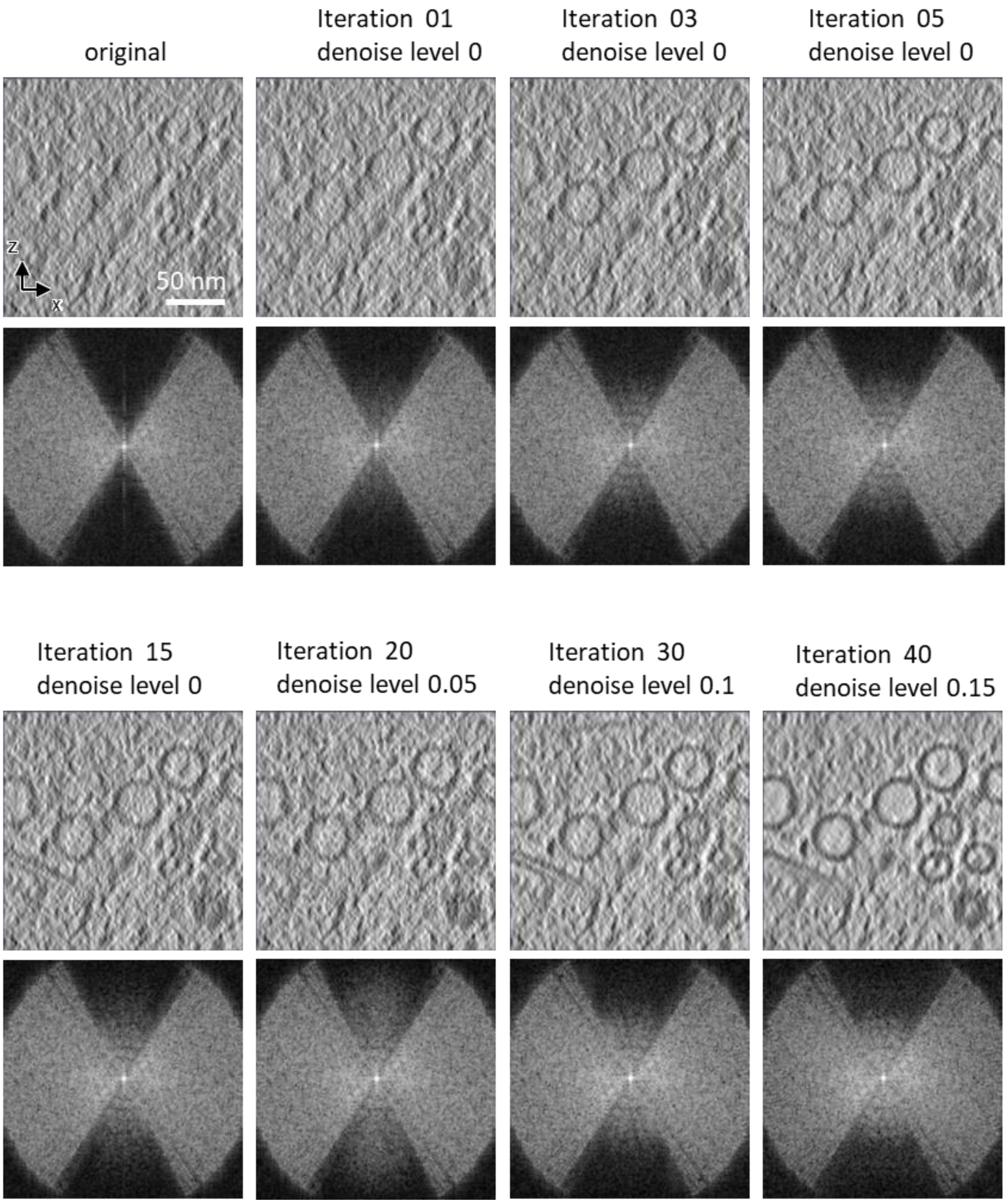

Supplementary Fig. 4 │ Iteratively filling the missing-wedge region. XZ slice views of subtomograms and corresponding power spectrum at different iterations in *Refine* step.

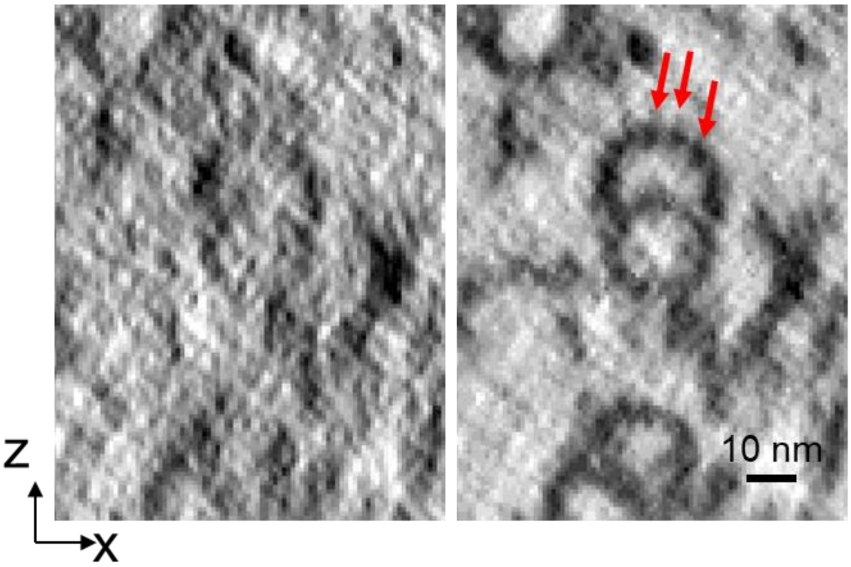

Supplementary Fig. 5 │ XZ slices of microtubule doublets in axoneme. Left: XZ slices of the SIRT reconstructions. Right: the slices of the corresponding tomogram generated by IsoNet. Red arrows indicate microtubule subunits.

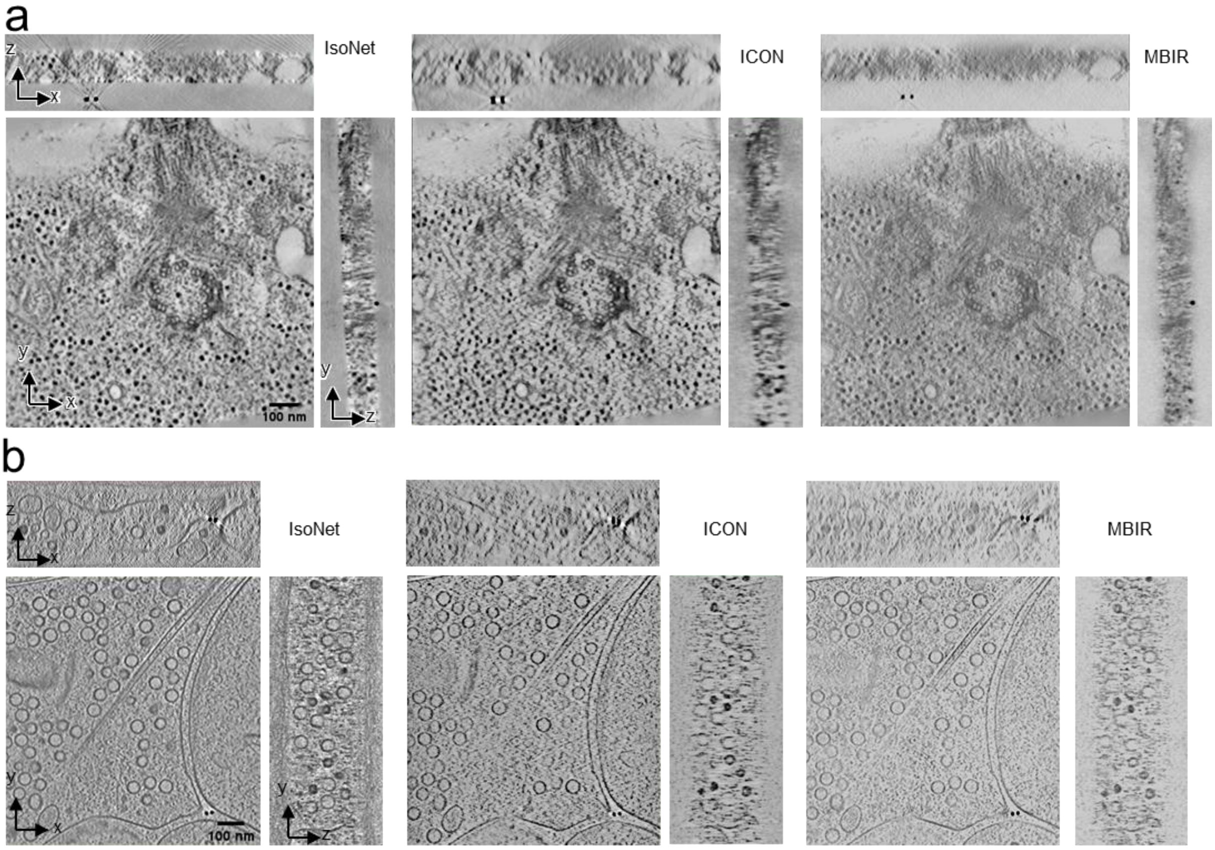

Supplementary Fig. 6 │ Compariasion the IsoNet reconstruction with ICON and MBIR reconstructions. a, Orthogonal slices of IsoNet, ICON and MBIR reconstructions of tilt series of thin sections provided in IMOD tutorial. **b,** Orthogonal slices of reconstructions of cryoET tomogram of neuronal synapses in cultured cells.

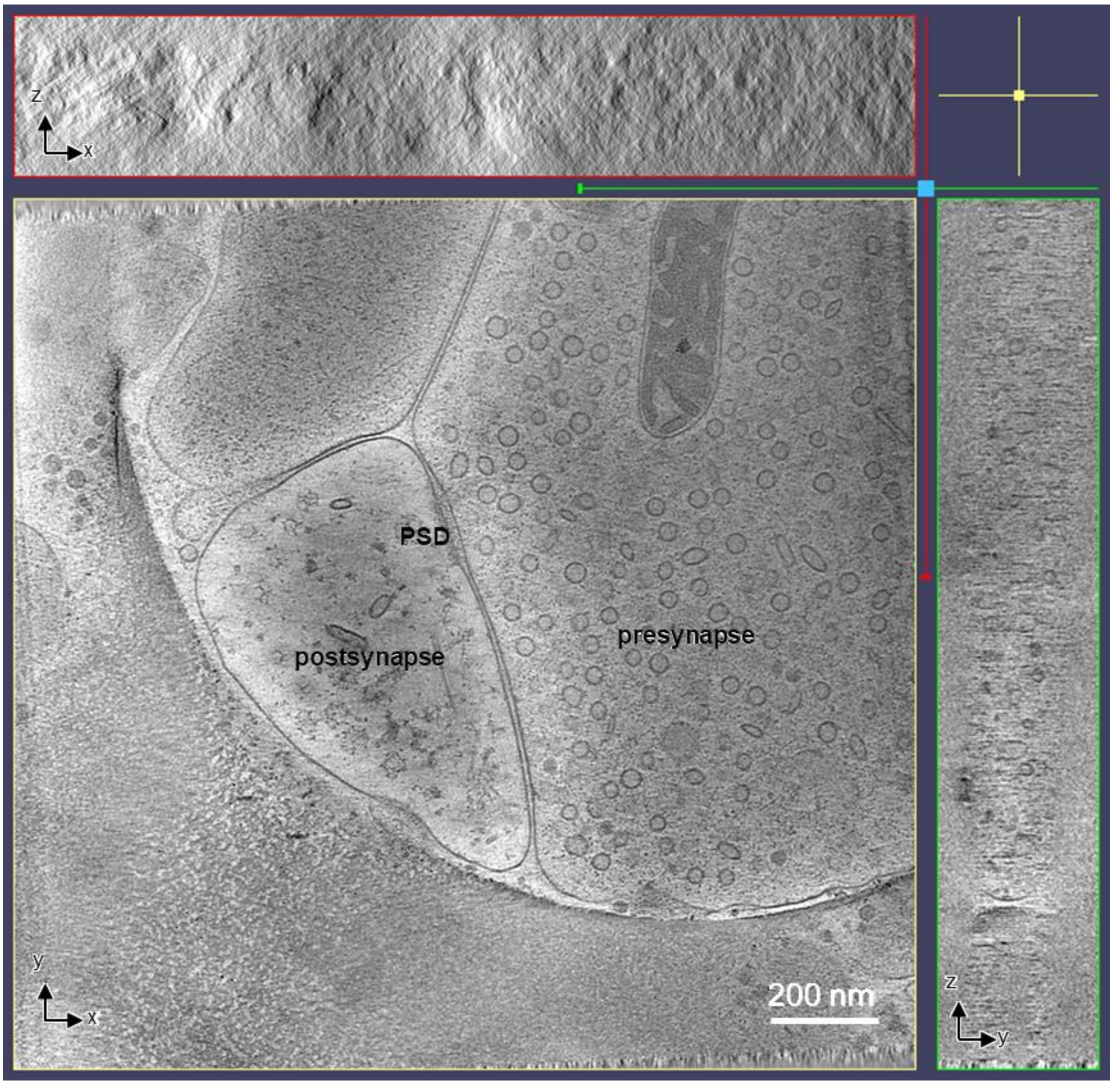

Supplementary Fig. 7 | Orthogonal views of the tomogram containing clathrin cages reconstructed with the SIRT algorithm in IMOD. PSD: postsynaptic density.

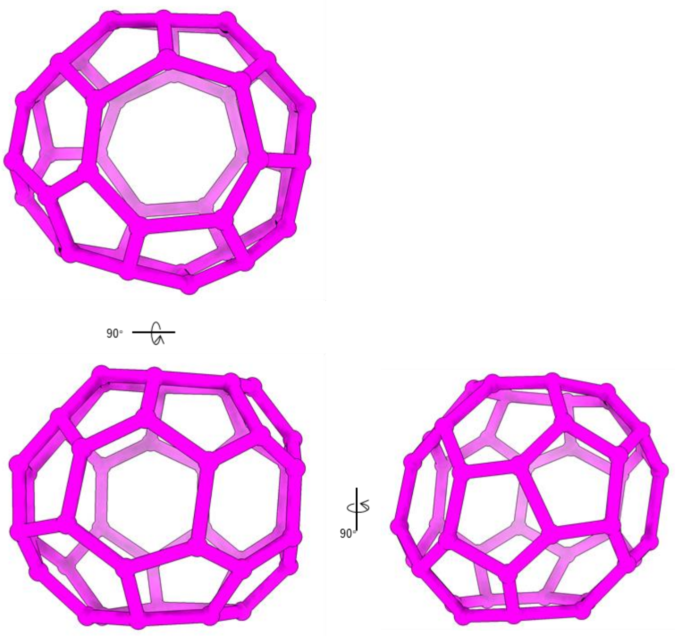

Supplementary Fig. 8 | 3D views for the shape of the heptagon containing clathrin cage
