# Supplementary material for: Isotropic reconstruction for electron tomography with deep learning
Source: Nat Commun. 2022 Oct 29;13:6482. doi: 10.1038/s41467-022-33957-8 (PMC9617606; doi:10.1038/s41467-022-33957-8)
Supplement: Supplementary file 3 — Description of Additional Supplementary Files [file 41467_2022_33957_MOESM3_ESM.docx]

**Description of Additional Supplementary Files**

**Supplementary Video 1** | 3D tomogram of HIV particles. This video shows 3D structures in an IsoNet generated tomogram. The tomogram density is sliced through three orthogonal directions.

**Supplementary Video 2** | 3D rendering of HIV particles. The HIV particles are rendered in yellow (fully embedded in ice) and blue (at air-water interface). The rest cryoEM density is shown in transparent gray.

**Supplementary Video 3** | 2D slices of a tomogram of a neuronal synapse. Left: IsoNet generated tomogram. Right: The original tomogram reconstructed with WBP.

**Supplementary Video 4** | 3D tomogram of a neuronal synapse. This video shows 3D structures in an IsoNet generated tomogram. The tomogram density is sliced through three orthogonal directions.

**Supplementary Video 5** | 3D rendering of the neuronal synapse.

**Supplementary Video 6** | 3D tomogram clathrin cages in a neuronal synapse. This video shows 3D structures of an IsoNet generated tomogram. The tomogram density is sliced through three orthogonal directions. Then, 3D rendering of clathrin cages is shown and rotated.
