# Supplementary material for: Isotropic reconstruction for electron tomography with deep learning
Source: Nat Commun. 2022 Oct 29;13:6482. doi: 10.1038/s41467-022-33957-8 (PMC9617606; doi:10.1038/s41467-022-33957-8)
Supplement: Supplementary file 10 — Reporting Summary [file 41467_2022_33957_MOESM10_ESM.pdf]

nature portfolio

Corresponding author(s): Z Hong Zhou and Guo-Qiang Bi

Last updated by author(s): Aug 23, 2022

# Reporting Summary

Nature Portfolio wishes to improve the reproducibility of the work that we publish. This form provides structure for consistency and transparency in reporting. For further information on Nature Portfolio policies, see our Editorial Policies and the Editorial Policy Checklist.

## Statistics

For all statistical analyses, confirm that the following items are present in the figure legend, table legend, main text, or Methods section.

| n/a | Confirmed | |
|---|---|---|
| x | ☐ | The exact sample size ($n$) for each experimental group/condition, given as a discrete number and unit of measurement |
| x | ☐ | A statement on whether measurements were taken from distinct samples or whether the same sample was measured repeatedly |
| x | ☐ | The statistical test(s) used AND whether they are one- or two-sided<br>*Only common tests should be described solely by name; describe more complex techniques in the Methods section.* |
| x | ☐ | A description of all covariates tested |
| x | ☐ | A description of any assumptions or corrections, such as tests of normality and adjustment for multiple comparisons |
| x | ☐ | A full description of the statistical parameters including central tendency (e.g. means) or other basic estimates (e.g. regression coefficient) AND variation (e.g. standard deviation) or associated estimates of uncertainty (e.g. confidence intervals) |
| x | ☐ | For null hypothesis testing, the test statistic (e.g. $F$, $t$, $r$) with confidence intervals, effect sizes, degrees of freedom and $P$ value noted<br>*Give P values as exact values whenever suitable.* |
| x | ☐ | For Bayesian analysis, information on the choice of priors and Markov chain Monte Carlo settings |
| x | ☐ | For hierarchical and complex designs, identification of the appropriate level for tests and full reporting of outcomes |
| x | ☐ | Estimates of effect sizes (e.g. Cohen's $d$, Pearson's $r$), indicating how they were calculated |

*Our web collection on statistics for biologists contains articles on many of the points above.*

## Software and code

Policy information about availability of computer code

| | |
|---|---|
| Data collection | SerialEM (v3.4.9): cryoET data collection on FEI Titan Krios scope |
| Data analysis | MotionCorr(v2): drift correction of cryoEM images in movie stack<br>CTFFIND (v4.1): defocus determination of cryoET images<br>IMOD (v4.8): cryoET tilt series alignment<br>NovaCTF (GitHub without version): CTF correction and tomogram reconstruction<br>UCSF ChimeraX (1.2.5): segmentation and 3D rendering<br>IsoNet(0.1 and 0.2): denoising and missing-wedge correction https://github.com/IsoNet-cryoET/IsoNet |

For manuscripts utilizing custom algorithms or software that are central to the research but not yet described in published literature, software must be made available to editors and reviewers. We strongly encourage code deposition in a community repository (e.g. GitHub). See the Nature Portfolio guidelines for submitting code & software for further information.

## Data

Policy information about availability of data

All manuscripts must include a data availability statement. This statement should provide the following information, where applicable:

- Accession codes, unique identifiers, or web links for publicly available datasets
- A description of any restrictions on data availability
- For clinical datasets or third party data, please ensure that the statement adheres to our policy

Dataset used in this study including 3 tomograms of HIV particles, one IsoNet corrected HIV tomogram, one original tomogram of a neuronal synapse with its IsoNet reconstruction, and 10 simulated apoferritin subtomograms is deposited into publicly available repository Figshare (https://figshare.com/articles/dataset/Dataset_to_reproduce_results_in_IsoNet_paper/20560443).

## Human research participants

Policy information about studies involving human research participants and Sex and Gender in Research.

| | |
|---|---|
| Reporting on sex and gender | N/A |
| Population characteristics | *Describe the covariate-relevant population characteristics of the human research participants (e.g. age, genotypic information, past and current diagnosis and treatment categories). If you filled out the behavioural & social sciences study design questions and have nothing to add here, write "See above."* |
| Recruitment | *Describe how participants were recruited. Outline any potential self-selection bias or other biases that may be present and how these are likely to impact results.* |
| Ethics oversight | *Identify the organization(s) that approved the study protocol.* |

Note that full information on the approval of the study protocol must also be provided in the manuscript.

# Field-specific reporting

Please select the one below that is the best fit for your research. If you are not sure, read the appropriate sections before making your selection.

☒ Life sciences ☐ Behavioural & social sciences ☐ Ecological, evolutionary & environmental sciences

For a reference copy of the document with all sections, see nature.com/documents/nr-reporting-summary-flat.pdf

# Life sciences study design

All studies must disclose on these points even when the disclosure is negative.

| | |
|---|---|
| Sample size | We used 10 in silico simulated subtomograms of apoferritin, 7 simulated tomograms of individual ribosome. 3 tomograms of HIV downloaded from EMPIAR-10164, 7 tomograms of ribosomes downloaded from EMPIAR-10045, 3 tomograms of Eukaryotic flagella, 5 tomograms of neuronal synapses. No statistical methods are used to determine the sample size. Sample size are choose by availability of the data and one or few tomograms are typical number of input for our IsoNet software. |
| Data exclusions | No data are excluded in this study. |
| Replication | Same results will be reproduced with same input using IsoNet software. |
| Randomization | This is not relevant to our study because samples are not allocated into groups |
| Blinding | This is not relevant to our study because samples are not allocated into groups. |

# Reporting for specific materials, systems and methods

We require information from authors about some types of materials, experimental systems and methods used in many studies. Here, indicate whether each material, system or method listed is relevant to your study. If you are not sure if a list item applies to your research, read the appropriate section before selecting a response.

## Materials & experimental systems

| n/a | Involved in the study |
| --- | --- |
| ☒ | ☐ Antibodies |
| ☒ | ☐ Eukaryotic cell lines |
| ☒ | ☐ Palaeontology and archaeology |
| ☒ | ☐ Animals and other organisms |
| ☒ | ☐ Clinical data |
| ☒ | ☐ Dual use research of concern |

## Methods

| n/a | Involved in the study |
| --- | --- |
| ☒ | ☐ ChIP-seq |
| ☒ | ☐ Flow cytometry |
| ☒ | ☐ MRI-based neuroimaging |
